# A predictive model for the risk of sepsis within 30 days of admission in patients with traumatic brain injury in the intensive care unit: a retrospective analysis based on MIMIC-IV database

**DOI:** 10.1186/s40001-023-01255-8

**Published:** 2023-08-18

**Authors:** Fangqi Hu, Jiaqiu Zhu, Sheng Zhang, Cheng Wang, Liangjia Zhang, Hui Zhou, Hui Shi

**Affiliations:** 1 Department of Neurosurgery, Lianyungang Clinical Medical College, Nanjing Medical University, Lianyungang, 222000 Jiangsu China; 2 Department of Neurosurgery, The Second People’s Hospital of Lianyungang City, Lianyungang, 222000 Jiangsu China; 3 Department of Neurosurgery, Huzhou Central Hospital, Huzhou, 313000 Zhejiang China

**Keywords:** Traumatic brain injury, Sepsis, Intensive care unit, MICMIC database, Nomogram

## Abstract

**Purpose:**

Traumatic brain injury (TBI) patients admitted to the intensive care unit (ICU) are at a high risk of infection and sepsis. However, there are few studies on predicting secondary sepsis in TBI patients in the ICU. This study aimed to build a prediction model for the risk of secondary sepsis in TBI patients in the ICU, and provide effective information for clinical diagnosis and treatment.

**Methods:**

Using the MIMIC IV database version 2.0 (Medical Information Mart for Intensive Care IV), we searched data on TBI patients admitted to ICU and considered them as a study cohort. The extracted data included patient demographic information, laboratory indicators, complications, and other clinical data. The study cohort was divided into a training cohort and a validation cohort. In the training cohort, variables were screened by LASSO (Least absolute shrinkage and selection operator) regression and stepwise Logistic regression to assess the predictive ability of each feature on the incidence of patients. The screened variables were included in the final Logistic regression model. Finally, the decision curve, calibration curve, and receiver operating character (ROC) were used to test the performance of the model.

**Results:**

Finally, a total of 1167 patients were included in the study, and these patients were randomly divided into the training (N = 817) and validation (N = 350) cohorts at a ratio of 7:3. In the training cohort, seven features were identified as key predictors of secondary sepsis in TBI patients in the ICU, including acute kidney injury (AKI), anemia, invasive ventilation, GCS (Glasgow Coma Scale) score, lactic acid, and blood calcium level, which were included in the final model. The areas under the ROC curve in the training cohort and the validation cohort were 0.756 and 0.711, respectively. The calibration curve and ROC curve show that the model has favorable predictive accuracy, while the decision curve shows that the model has favorable clinical benefits with good and robust predictive efficiency.

**Conclusion:**

We have developed a nomogram model for predicting secondary sepsis in TBI patients admitted to the ICU, which can provide useful predictive information for clinical decision-making.

## Background

Traumatic brain injury (TBI) refers to impaired brain function or other brain pathological changes [1] caused by external forces, including concussion and traumatic brain hernia. At present, its incidence rate is the highest among all common nervous system diseases, and every year, 50 million to 60 million new TBI cases are reported worldwide, causing a huge public health burden [2]. In 2016, the Third International Consensus Definitions for Sepsis and Septic Shock (Sepsis 3.0) defined sepsis as a physiologically, pathologically and biochemically abnormal syndrome induced by infection, which is accompanied by acute organ dysfunction [3]. In 2017, the number of sepsis patients was estimated to be 48.9 million, and the deaths of sepsis exceeded 11 million, accounting for 19.7% of the annual death toll [4]. TBI patients in ICU need to receive more comprehensive treatment, including continuous intracranial pressure detection, decompressive craniectomy, early enteral nutrition, auxiliary ventilation, and fluid therapy to maintain arterial pressure and internal organ perfusion [5]. They are at a high risk of drug-resistant bacteria infection and secondary sepsis [6]. The high-risk factors for secondary sepsis among TBI patients in ICU are as follows: 1. hospital-acquired pneumonia (HAP) is the most common complication of long-term bed rest [7]; 2. because of long-term consciousness disorder and neurological deficit, TBI patients in ICU require long-term nursing and various invasive operations, such as tracheotomy, mechanical assisted ventilation [8], emergency operation, nasogastric tube [9], urinary catheter and deep vein catheterization [10], all of which are all high-risk factors for infection; 3. secondary stress ulcer, early epilepsy, and deep vein thrombosis after TBI lead to disease progression and prolong hospitalization [11]. TBI patients admitted to ICU are in a state of consciousness disturbance for a long time and unable to feed back condition immediately. Therefore, the occurrence of infection and sepsis in these patients is usually insidious. As a result, it is usually delayed and challenging for clinicians to identify secondary sepsis in such patients.

Early identification of sepsis in TBI patients in the ICU is necessary. Clinical prediction models can provide effective information for clinicians to identify high-risk patients, make clinical decisions, and take countermeasures. However, there are few studies on the prediction of sepsis in TBI patients in the ICU. Hence, this study established a model for predicting the occurrence of sepsis in TBI patients in the ICU. The model has good prediction performance and can provide effective prediction information.

## Methods

### Data source

The data on patients diagnosed with TBI and admitted to ICU were extracted from MIMIC-IV 2.0 database (https://physionet.org/content/mimiciv/2.0/), and the patients with intracranial injury in the database were identified based on ICD 9 and ICD 10 (ICD 10: S06; ICD 9: 85). To improve the simplicity of the model, we chose variables that were readily available in the clinic. The collected data include patient demographic data (gender, marital status, race, age), complications (acidosis, acute kidney injury, anemia, atrial fibrillation, depressive, diabetes, esophageal reflux, heart failure, hyperlipidemia, hypertension, thrombocytopenia, toxic encephalopathy, urinary tract infection), drug treatment information (dopamine, epinephrine, norepinephrine), operative procedure information (invasive ventilation, nasal gastric tube, urinary catheter), and laboratory indicators (lactate, basophils, eosinophils, lymphocytes, monocytes, neutrophils, anion gap, bicarbonate, calcium, creatinine, urea nitrogen, international normalized ratio, prothrombin time, activated partial thromboplastin time, hematocrit, hemoglobin, mean corpuscular hemoglobin, mean corpuscular hemoglobin concentration, mean corpuscular volume, platelets, red blood cell, red blood cell distribution width, white blood cells). For patients with multiple admissions, the first hospitalization data were used. For data from multiple examinations, data from the first examination within 24 h of admission were used. To prevent reverse causality, information on surgical procedures and medications after a patient developed sepsis was considered invalid and was not included in the analysis. Sepsis was diagnosed according to Sepsis 3.0 [3]. Sepsis events after 30 days of admission were not included in the analysis. Those cases that were not admitted to ICU or had sepsis before admission to ICU and whose data were missed were excluded. Informed consent of patients was not required for this study because the database was approved by the Institutional Review Committee of MIT and Beth Israel Deaconess Medical Center.

### Statistical analysis

The ‘‘createDataPartition’’ function of the caret software package was used to group patients into the training and validation cohorts at a ratio of 7:3, so that the outcome events were randomly distributed in the two cohorts. In order to prevent over-fitting of the model, most of the data were used to train the model to ensure its accuracy, while a small part of the data were used for validation. Variables were described in the training dataset and validation dataset, respectively. Categorical variables were described as percentiles (%); continuous variables of non-normal distribution were displayed as medians and quartiles, and continuous variables of normal distribution were expressed as mean and standard deviation (mean (S.E.)). The chi-square test was used to compare the differences between categorical variables, and the t-test or nonparametric test was used to compare the differences between two groups of continuous variables. In the training cohort, LASSO regression and stepwise Logistic regression based on AIC (Akaike Information Criterion) were used for feature selection. Statistically significant variables (P < 0.05) were identified as independent risk factors and were included in the final logistic regression model, and a corresponding nomogram was plotted. The area under the ROC curve (AUC) was used to assess the prediction accuracy of the model; calibration curve was used to assess the consistency between the predicted value of the model and the actual value, and decision curve was used to analyze the clinical benefits of the model. Tableone software package was used for data description; glmnet software package was used for LASSO regression analysis; rms software package was used for plotting the nomogram and calibration curve, and pROC software package was used for plotting ROC curve. R 4.2.1 (https://www.r-project.org) was used for all statistical analysis. A two-sided *P* value < 0.05 was considered statistically significant. This study was designed and analyzed with reference to the TRIPOD (Transparent Reporting of a multivariable prediction model for Individual Prognosis or Diagnosis) statement [12].

## Results

### Characteristics of the study cohort

**Fig. 1:**
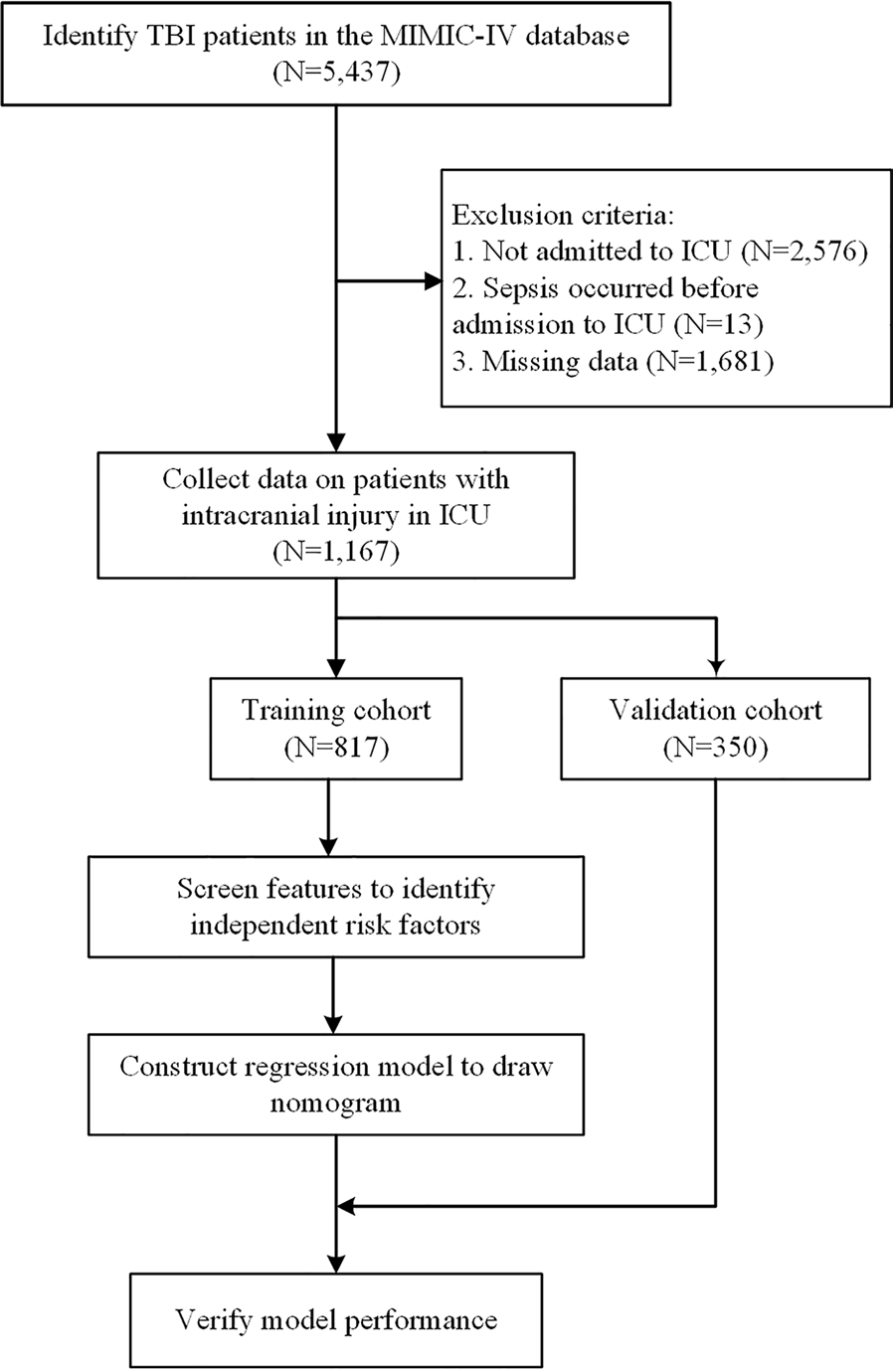
Flowchart of the study

A total of 5437 TBI patients were identified from the database. After those patients with missing data (N = 1681), not admitted to ICU (N = 2576), and diagnosed with sepsis before admission to ICU (N = 13) were excluded, 1167 patients (535 with secondary sepsis) were included in the study, including 817 (385 secondary sepsis) in the training cohort and 350 (150 with secondary sepsis) in the validation cohort. (Fig. 1) The study cohort was predominantly male (study cohort: 63.3%; training cohort: 64.1%; validation cohort: 61.4%). The median ages of the study cohort, training cohort, and validation cohort were 66, 65, and 66 years, respectively. Table 1 summarizes the demographic and clinical data of the study cohort. The variables in the training cohort and validation cohort were comparable with no statistically significant difference (*P* < 0.05).

**Table 1:** Characteristics description of patients

	Overall	Training set	Validation set	p
N	1167	817	350	
Status = 1 (%)	535 (45.8)	385 (47.1)	150 (42.9)	0.202
Gender = Male (%)	739 (63.3)	524 (64.1)	215 (61.4)	0.416
Marital status (%)				0.239
Divorced	54 (4.6)	31 (3.8)	23 (6.6)	
Married	409 (35.0)	288 (35.3)	121 (34.6)	
Other	223 (19.1)	163 (20.0)	60 (17.1)	
Single	319 (27.3)	225 (27.5)	94 (26.9)	
Window	162 (13.9)	110 (13.5)	52 (14.9)	
Race (%)				0.069
Asian	33 (2.8)	17 (2.1)	16 (4.6)	
Black	61 (5.2)	44 (5.4)	17 (4.9)	
Other	354 (30.3)	258 (31.6)	96 (27.4)	
White	719 (61.6)	498 (61.0)	221 (63.1)	
Acidosis = Yes (%)	194 (16.6)	131 (16.0)	63 (18.0)	0.459
AKI = Yes (%)	273 (23.4)	190 (23.3)	83 (23.7)	0.925
Anemia = Yes (%)	480 (41.1)	339 (41.5)	141 (40.3)	0.75
Atrial fibrillation = Yes (%)	170 (14.6)	122 (14.9)	48 (13.7)	0.653
Depressive = Yes (%)	210 (18.0)	145 (17.7)	65 (18.6)	0.801
Diabetes = Yes (%)	281 (24.1)	200 (24.5)	81 (23.1)	0.678
Esophageal reflux = Yes (%)	214 (18.3)	155 (19.0)	59 (16.9)	0.44
Heart failure = Yes (%)	213 (18.3)	146 (17.9)	67 (19.1)	0.665
Hyperlipidemia = Yes (%)	359 (30.8)	241 (29.5)	118 (33.7)	0.174
Hypertension = Yes (%)	613 (52.5)	426 (52.1)	187 (53.4)	0.734
Thrombocytopenia = Yes (%)	186 (15.9)	130 (15.9)	56 (16.0)	1
Toxic encephalopathy = Yes (%)	135 (11.6)	89 (10.9)	46 (13.1)	0.317
Urinary tract infection = Yes (%)	297 (25.4)	205 (25.1)	92 (26.3)	0.722
Dopamine = Yes (%)	9 (0.8)	6 (0.7)	3 (0.9)	1
Epinephrine = Yes (%)	13 (1.1)	10 (1.2)	3 (0.9)	0.808
Norepinephrine = Yes (%)	70 (6.0)	51 (6.2)	19 (5.4)	0.688
Invasive ventilation = Yes (%)	417 (35.7)	301 (36.8)	116 (33.1)	0.254
NGT = Yes (%)	45 (3.9)	27 (3.3)	18 (5.1)	0.184
Urinary catheter = Yes (%)	96 (8.2)	55 (6.7)	41 (11.7)	0.006
GCS score (%)				0.498
13–15	692 (59.3)	490 (60.0)	202 (57.7)	
3–5	91 (7.8)	67 (8.2)	24 (6.9)	
6–8	147 (12.6)	103 (12.6)	44 (12.6)	
9–12	237 (20.3)	157 (19.2)	80 (22.9)	
Age (median [IQR])	66.00 [49.00, 80.00]	65.00 [50.00, 80.00]	66.00 [47.25, 80.75]	0.732
Lactate (mmol/L) (median [IQR])	1.90 [1.40, 2.95]	2.00 [1.40, 3.00]	1.90 [1.40, 2.77]	0.296
Basophils (%) (median [IQR])	0.30 [0.20, 0.50]	0.30 [0.20, 0.50]	0.30 [0.20, 0.50]	0.216
Eosinophils (%) (median [IQR])	0.60 [0.10, 1.50]	0.60 [0.10, 1.70]	0.60 [0.20, 1.30]	0.615
Lymphocytes (%) (median [IQR])	12.20 [7.70, 19.70]	12.20 [7.60, 20.00]	12.45 [7.70, 18.40]	0.976
Monocytes (%) (median [IQR])	5.70 [4.05, 7.70]	5.70 [4.10, 7.60]	5.60 [4.03, 7.70]	0.857
Neutrophils (%) (median [IQR])	78.90 [69.70, 85.50]	78.60 [69.20, 85.60]	79.35 [70.73, 85.18]	0.917
Anion gap (mEq/L) (median [IQR])	16.00 [13.00, 18.00]	16.00 [13.00, 18.00]	15.00 [13.00, 18.00]	0.262
Bicarbonate (mEq/L) (median [IQR])	23.00 [21.00, 26.00]	23.00 [21.00, 26.00]	23.00 [21.00, 26.00]	0.722
Calcium (mg/dL) (median [IQR])	8.60 [8.10, 9.10]	8.60 [8.10, 9.10]	8.60 [8.20, 9.10]	0.606
Creatinine (mg/dL) (median [IQR])	0.90 [0.75, 1.20]	1.00 [0.80, 1.20]	0.90 [0.70, 1.10]	0.095
Urea nitrogen (mg/dL) (median [IQR])	17.00 [12.00, 23.00]	17.00 [13.00, 23.00]	16.00 [12.00, 22.00]	0.271
INR (median [IQR])	1.10 [1.00, 1.30]	1.10 [1.00, 1.30]	1.10 [1.00, 1.20]	0.324
PT (s) (median [IQR])	12.30 [11.40, 14.05]	12.40 [11.40, 14.20]	12.30 [11.40, 13.60]	0.24
PTT (s) (median [IQR])	27.60 [24.90, 31.30]	27.80 [25.00, 31.70]	27.20 [24.83, 30.67]	0.114
Hematocrit (%) (median [IQR])	38.00 [33.80, 41.70]	37.80 [33.40, 41.60]	38.40 [34.92, 42.00]	0.104
Hemoglobin (g/dL) (median [IQR])	12.70 [11.20, 14.00]	12.70 [11.10, 14.00]	12.90 [11.60, 14.00]	0.057
MCH (pg) (median [IQR])	30.80 [29.50, 32.20]	30.70 [29.40, 32.20]	30.85 [29.52, 32.10]	0.851
MCHC (g/dL) (median [IQR])	33.40 [32.40, 34.30]	33.40 [32.40, 34.30]	33.50 [32.50, 34.38]	0.464
MCV (fL) (median [IQR])	92.00 [88.00, 96.00]	92.00 [88.00, 96.00]	92.00 [89.00, 96.00]	0.76
Platelets (K/µL) (median [IQR])	211.00 [168.50, 267.00]	213.00 [169.00, 269.00]	208.00 [166.25, 254.75]	0.145
RBC (m/µL) (median [IQR])	4.13 [3.64, 4.56]	4.11 [3.60, 4.56]	4.18 [3.73, 4.53]	0.169
RDW (%) (median [IQR])	13.60 [12.90, 14.60]	13.60 [12.90, 14.60]	13.50 [12.90, 14.60]	0.409
WBC (K/µL) (median [IQR])	10.70 [7.80, 14.80]	10.80 [7.80, 15.00]	10.50 [7.73, 14.47]	0.318

*AKI* acute kidney injure, *INR* international normalized ratio, *PT* prothrombin time, *PTT* activated partial thromboplastin time, *MCH* mean corpuscular hemoglobin, *MCHC* mean corpuscular hemoglobin concentration, *MCV* mean corpuscular volume, *RBC* red blood cell, *RDW* red blood cell distribution width, *WBC* white blood cells, *GCS* Glasgow coma scale, *NGT* nasal gastric tube

### Results of feature selection

**Fig. 2:**
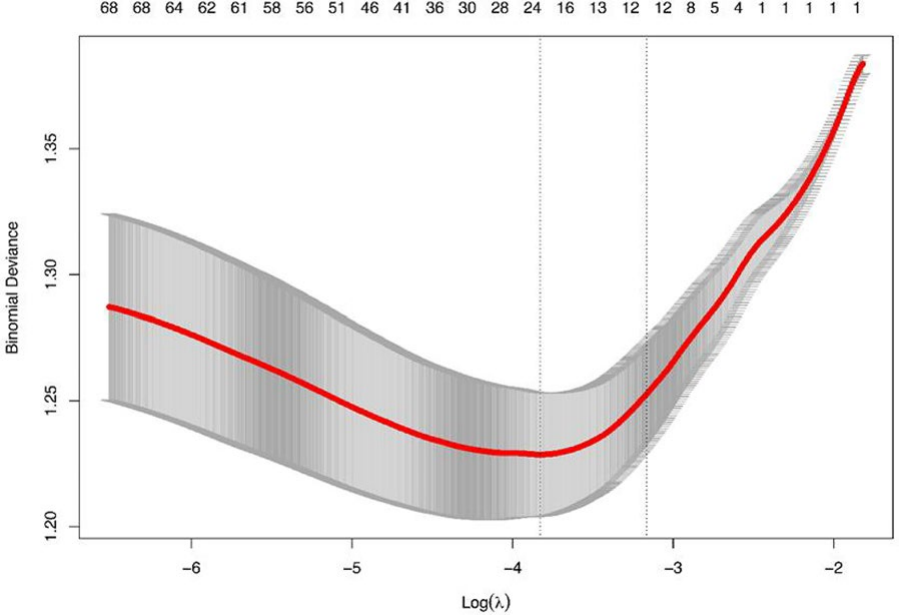
Lasso regression variable trajectories

The feature selection was conducted by using LASSO regression and stepwise Logistic regression. Figure 2 and Table 2 show the results of LASSO regression screening variables, and the x-coordinate at the top of Fig. 2 indicates the number of variables (dummy variables). The results showed that when λ was taken as the minimum value (0.02172893), 13 variables (Tables 2, 3) of 48 variables passed the screening and were included in the model (i.e., non-zero variables).

**Table 2:** The screening results of Lasso regression

		Coefficients
	(Intercept)	3.0307
Gender	Female	0.0000
	Male	0.0000
Marital status	Divorced	0.0000
	Married	0.0000
	Other	0.2493
	Single	0.0000
	Window	0.0000
Race	Asian	0.0000
	Black	0.0000
	Other	0.0000
	White	0.0000
Acidosis	No	0.0000
	Yes	0.0000
AKI	No	− 0.1842
	Yes	0.0000
Anemia	No	− 0.2749
	Yes	0.0000
Atrial fibrillation	No	0.0000
	Yes	0.0000
Depressive	No	0.0000
	Yes	0.0000
Diabetes	No	0.0000
	Yes	0.0000
Esophageal reflux	No	0.0000
	Yes	0.0000
Heart failure	No	0.0000
	Yes	0.0000
Hyperlipidemia	No	0.0000
	Yes	0.0000
Hypertension	No	0.0000
	Yes	0.0000
Thrombocytopenia	No	0.0000
	Yes	0.0000
Toxic encephalopathy	No	− 0.0049
	Yes	0.0000
Urinary tract infection	No	− 0.0411
	Yes	0.0000
Dopamine	No	0.0000
	Yes	0.0000
Epinephrine	No	0.0707
	Yes	0.0000
Norepinephrine	No	0.0000
	Yes	0.0000
Invasive ventilation	No	-0.2918
	Yes	0.0000
NGT	No	0.0000
	Yes	0.0000
Urinary catheter	No	0.0000
	Yes	0.0000
GCS	13–15	− 0.8031
	3–5	0.5318
	6–8	0.6161
	9–12	0.0000
Age		− 0.0008
Lactate		0.0455
Basophils		− 0.0954
Eosinophils		0.0000
Lymphocytes		0.0000
Monocytes		0.0004
Neutrophils		0.0000
Anion gap		0.0000
Bicarbonate		− 0.0262
Calcium		− 0.2159
Creatinine		0.0000
Urea nitrogen		0.0000
INR		0.0000
PT		0.0000
PTT		0.0000
Hematocrit		0.0000
Hemoglobin		0.0000
MCH		0.0000
MCHC		0.0000
MCV		0.0000
Platelets		0.0000
RBC		0.0000
RDW		0.0000
WBC		0.0000

**Table 3:** Multivariate regression model based on LASSO regression and stepwise logistic regression analysis results

Characteristic	OR	95% CI^1^	p-value
AKI			
No	–	–	
Yes	1.09	1.01, 1.17	0.033
Anemia			
No	–	–	
Yes	1.09	1.02, 1.17	0.008
Invasive ventilation			
No	–	–	
Yes	1.11	1.03, 1.19	0.003
GCS			
13–15	–	–	
3–5	1.47	1.30, 1.65	< 0.001
6–8	1.50	1.36, 1.65	< 0.001
9–12	1.21	1.12, 1.32	< 0.001
Lactate	1.02	1.01, 1.04	0.005
Calcium	0.93	0.89, 0.96	< 0.001

^1^*CI* confidence interval

In order to ensure the simplicity of the model, these 13 variables were further screened by using stepwise Logistic regression based on AIC screening. The final results showed that AKI, anemia, invasive ventilation, GCS score, lactic acid, and serum calcium levels were independent predictors of secondary sepsis in TBI patients in the ICU. In addition, the results showed that patients with AKI, anemia, moderate and severe disturbance of consciousness (GCS score ≤ 12), and invasive ventilation had a higher risk of sepsis.

### Construction and validation of nomogram

**Fig. 3:**
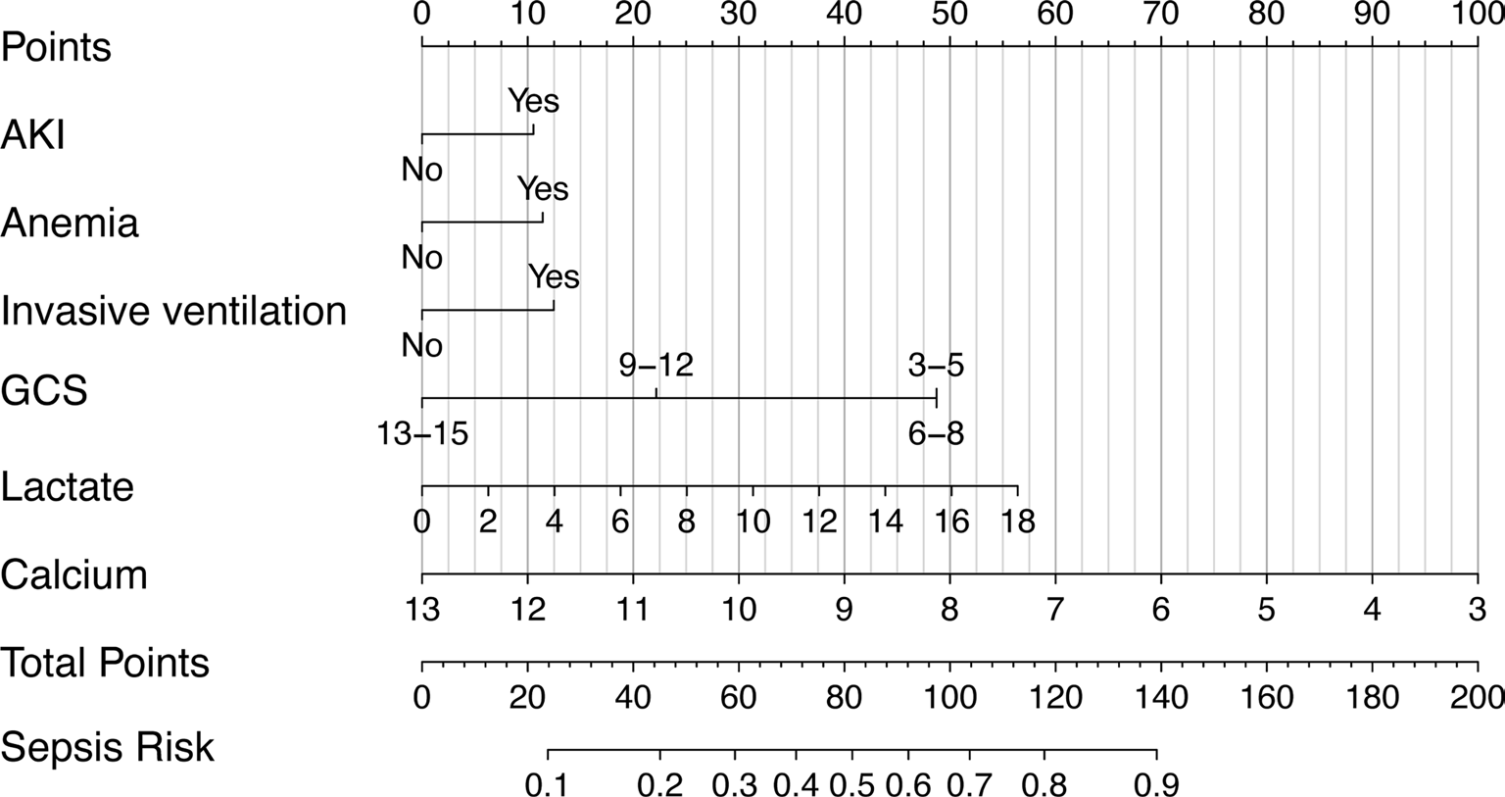
The predictive nomogram for the incidence of sepsis in patients with traumatic brain injury

**Fig. 4:**
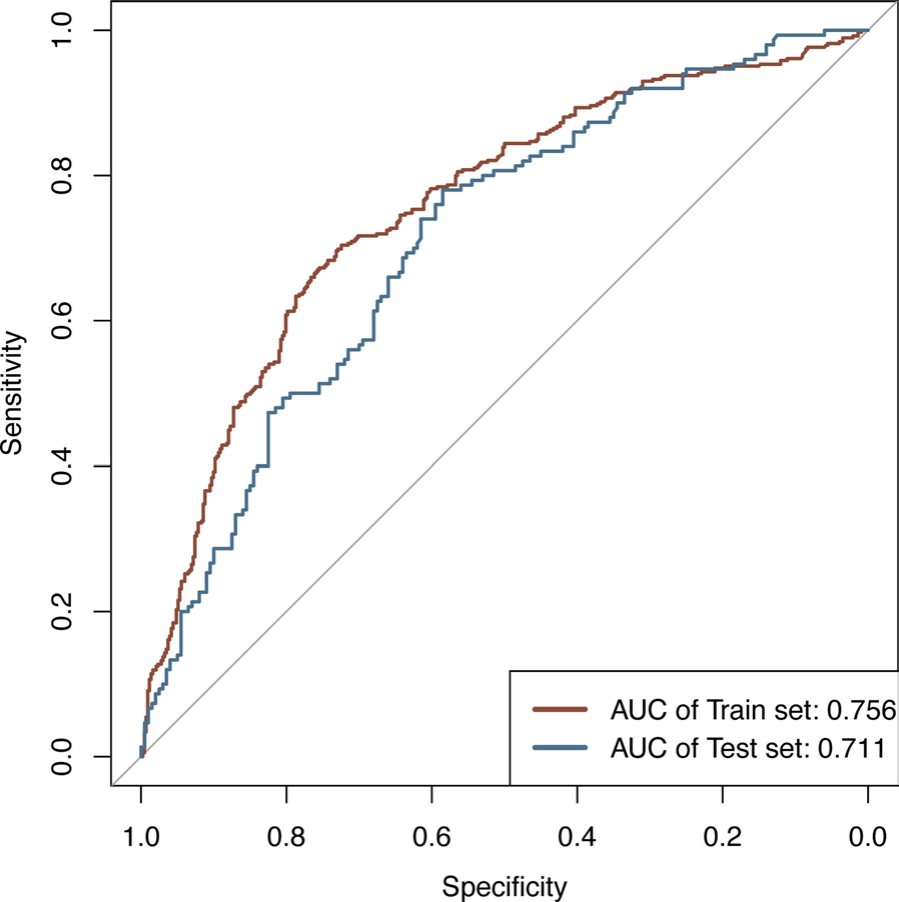
The results of ROC curve analysis in the training set and the validation set

**Fig. 5:**
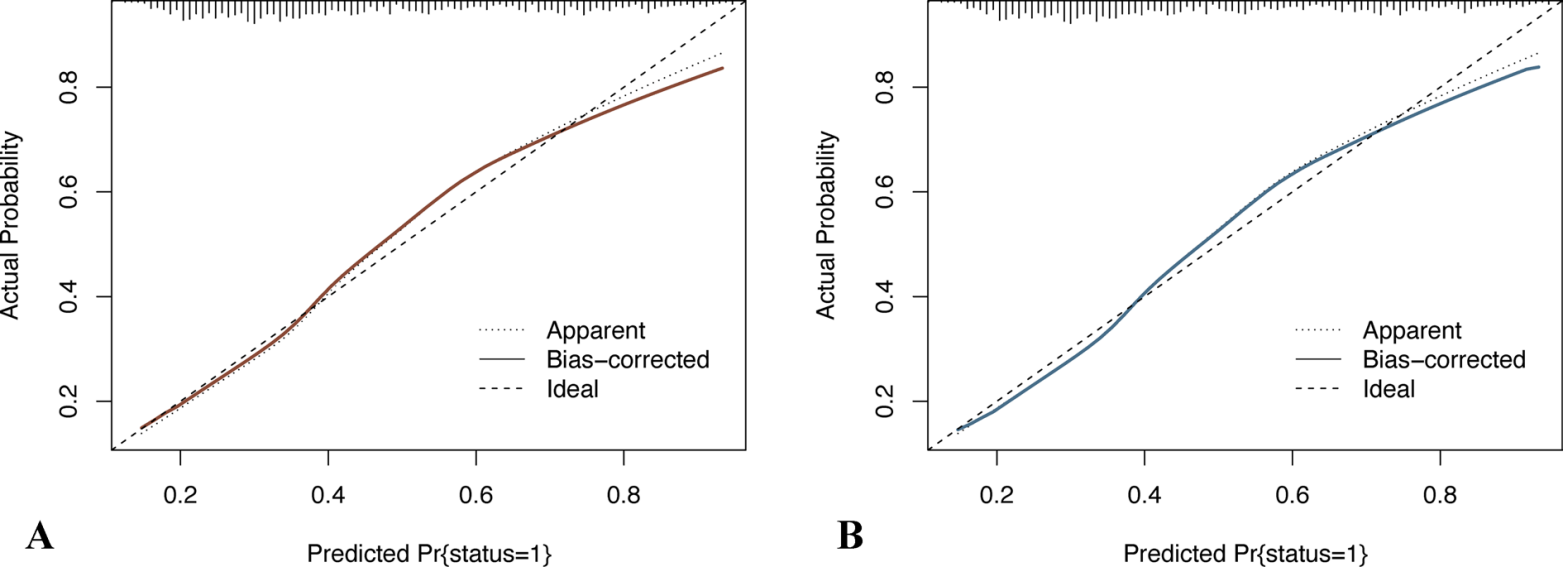
**A** The results of the calibration curve analysis in the training set; **B** The results of the calibration curve analysis in the validation set

**Fig. 6:**
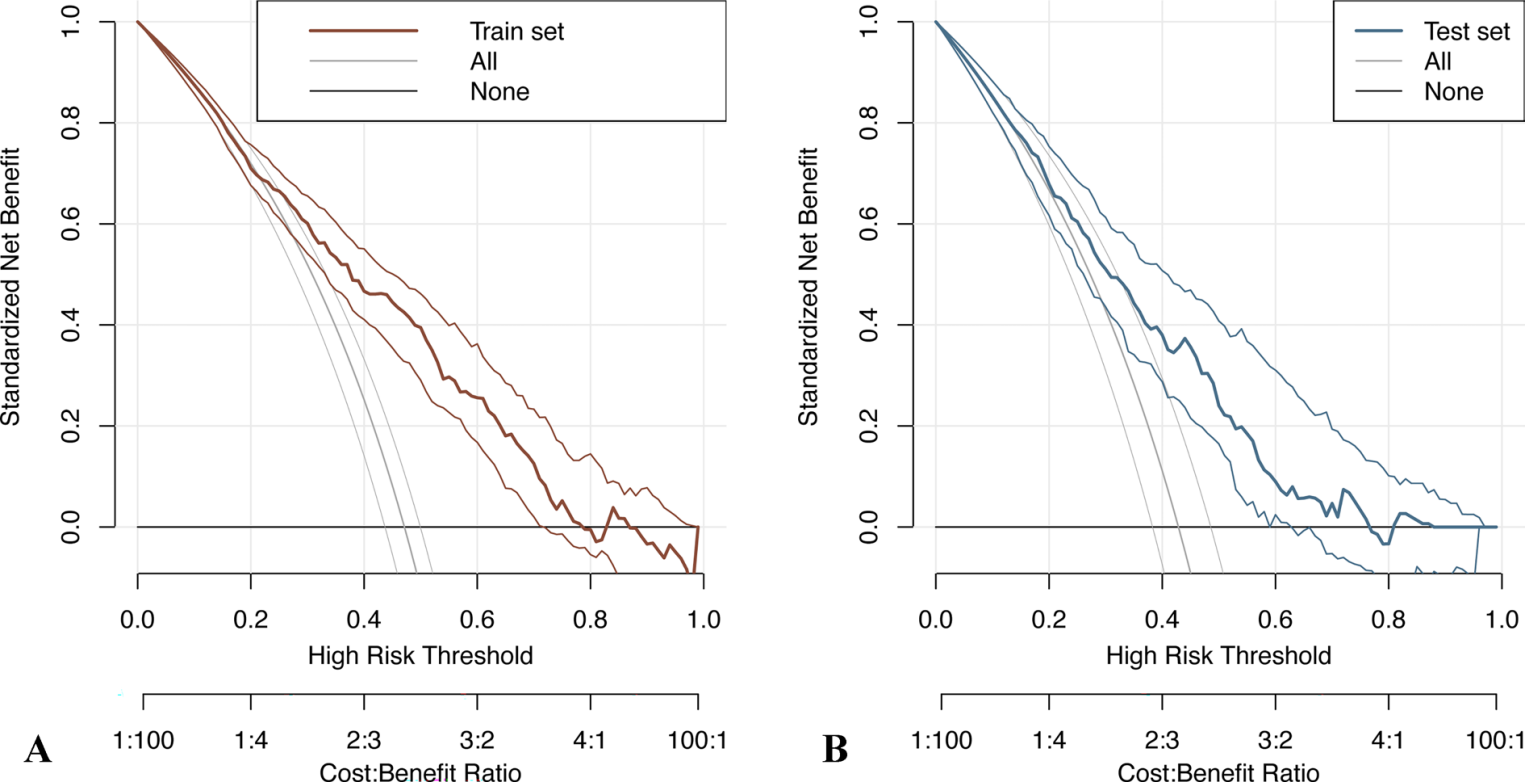
(**A**) The results of the decision curve analysis in the training set; **B** The results of the decision curve analysis in the validation set

Based on the screened features, a logistic regression model was constructed, and a nomogram was plotted (Fig. 3). Total points can be obtained by adding the scores of each variable in the nomogram, and the probability corresponding to the total score of the nomogram in the predictor (‘‘Sepsis Risk’’) is the probability of secondary sepsis in the patient. ROC curve, calibration curve, and decision curve were plotted to verify the model. ROC curve analysis results show that the AUC was 0.756 and 0.711 in the training cohort and the validation cohort (Fig. 4), respectively, indicating that the model had good discrimination ability. In the calibration curve, the y-coordinate indicates the actual incidence probability in the study cohort, and the x-coordinate indicates the estimated probability of the model. As shown in Fig. 5, the estimated probability has a high coincidence with the actual values, suggesting good consistency. In the clinical decision curve, the gray diagonal line indicates that all patients have received interventions; gray parallel line indicates that no patients have received intervention, and red (Fig. 6A) and blue curves (Fig. 6B) indicate the clinical benefits of the nomogram in the training cohort and validation cohort respectively. As shown in Fig. 6, our model has considerable net benefits in both cohorts.

## Discussion

The study subjects in this study were TBI patients admitted to ICU. Based on these patients’ demographic information, laboratory test indicators, and complications, a nomogram for predicting the risk of secondary sepsis in TBI patients was plotted. The results of feature selection showed that AKI, anemia, invasive ventilation, GCS score, lactic acid, and serum calcium level were important predictors of secondary sepsis in TBI patients in the ICU. The AUC of the nomogram model based on the above variables is greater than 0.7 in both training validation cohorts, indicating favorable prediction accuracy.

Sepsis is a life-threatening organ dysfunction caused by disordered body responses to infection [3]. The severity of a patient's condition can be assessed in a standardized manner by using the Sequential Organ Failure Assessment (SOFA), which can reveal the direct relation between sepsis and mortality [13, 14]. It is estimated that sepsis affects 19–48.9 million individuals and leads to over 20% global deaths as a main killer every year [4, 15, 16]. Early identification and treatment of sepsis can significantly reduce in-hospital mortality [17, 18]. In recent years, some prediction models for the risk of secondary sepsis in patients have been studied. Epic Sepsis model (ESM) is a sepsis early warning system widely used in the US. Andrew Wong et al. [19] conducted external verification based on a cohort of 27,697 people, but the final AUC was 0.63, showing poor discrimination and calibration ability. Their results suggested that more specific predictors, including onset time, should be included in such models. At present, various machine learning models for sepsis prediction have been constructed [20]. Dong Wang et al. developed a prediction model based on 4449 infected patients in ICU by supervised learning method to predict the occurrence of sepsis, and the AUC of this model can reach 0.91 [21]. Alireza Rafiei et al. developed a sepsis prediction model by using the convolution neural network, which included the onset time of sepsis, with an AUC of greater than 0.8 [22]. However, machine learning models have a ‘‘black box’’ effect, because it neither clearly shows the prediction process nor quantifies the prediction efficiency of each index. As such, clinicians do not trust these prediction results [23]. Many previous studies have utilized ICD code as sepsis diagnosis criteria [20], but this practice may produce unreliable results [24, 25].

Therefore, we took a specific population (i.e., TBI patients) as subjects, whose onset risks were predicted specifically by using up-to-date international consensus as the diagnosis criteria in this study. Besides, we constructed a nomogram model based on stepwise Logistic regression. As a visualized model, a nomogram can quantify the influence of each prediction variable on the results and offer practical explanations [26]. The nomogram model is simple and applicable and facilitates better and more efficient clinical decisions.

Our results showed that AKI, anemia, invasive ventilation, GCS score, lactic acid, and serum calcium level were significant predictors of secondary sepsis in TBI patients in the ICU. Furthermore, TBI patients with AKI have a higher risk of sepsis. For TBI patients, post-traumatic sympathetic nervous system activation, increased plasma catecholamine level, elevated systolic blood pressure, low blood volume, cytokine cascade reaction, and osmotic therapy of intracranial hypertension will bring a higher risk of kidney injury [27, 28]. The changes in intrathoracic pressure related to mechanical ventilation disrupt the systemic hemodynamics, resulting in biological damage such as decreased glomerular filtration rate, decreased creatinine clearance rate, and apoptosis of renal epithelial cells [29]. AKI leads to metabolic dysfunction such as electrolyte disorder and acid–base disorder, thus impairing neutrophil functions and weakening infection-eliminating ability in patients [29]. Animal experiments have confirmed that the lung recruitment of neutrophils with renal insufficiency is significantly weakened compared with that of normal neutrophils [30]. The insufficiency is associated with the change of surface expression of L-selectin, directly leading to higher bacterial loads and impaired pulmonary oxygenation function and thus deteriorating bacterial pneumonia.

The risk of acute lung injury and infection in TBI patients is increased due to post-traumatic autoimmune and lung immune damage, neurogenic pulmonary edema, and impaired lung protective mechanisms following disturbance of consciousness [31]. Studies have shown that 20–25% of TBI patients have respiratory failure, which is related to an increased oxygen demand or the ratio of arterial oxygen partial pressure to respiratory oxygen partial pressure (PaO_2_/FiO_2_ < 300) [26]. Ventilator-associated pneumonia (VAP) is one of the most common complications in TBI patients, with an incidence rate ranging from 23 to 60%. Tracheal intubation, tracheotomy, and ventilation support will increase the incidence of VAP [32]. The use of antibiotics increases the likelihood of drug-resistant bacteria infection, thereby resulting in a higher risk of secondary sepsis in TBI patients.

Lactic acid is a commonly used biological marker for the diagnosis and prognosis of sepsis [33], and serves as a sign of tissue hypoxia. For sepsis patients with normal blood pressure, lactic acid of more than 4 mmol/L is independently associated with higher mortality. The patients who have moderate hyperlactacidemia (2–4 mmol/L and even high value (1.4–2.3 mmol/L) in the normal range) have a worse prognosis than those with normal lactic acid [34]. Acidic extracellular environment will reduce myocardial contractility, cardiac output, blood pressure, and tissue perfusion, thereby leading to arrhythmia and weakening cardiovascular response to catecholamine [35], while high-dose catecholamine will aggravate hyperlactacidemia by reducing tissue perfusion or over-stimulating β2-adrenergic receptor. Therefore, tissue perfusion should be restored in the early stage of hyperlactacidemia to prevent further progression of the disease.

Due to fluid dilution caused by intravenous fluid resuscitation and traumatic bleeding (preoperative and perioperative periods), anemia is very common in TBI patients, especially in moderate and severe TBI patients [36]. It also aggravates tissue hypoxia and is more likely to lead to acute bacterial infection, especially Gram-positive bacterial infection [37]. Higher blood oxygen saturation and hemoglobin level and lower lactic acid level can significantly reduce the risk of death in patients with sepsis or septic shock [17].

Moreover, our results show that consciousness disturbance is one of the risk factors for sepsis, which is consistent with our hypothesis. TBI patients face an increased risk of HAP due to changes in mental state, dysphagia, vomiting, cough reflex, and secretion clearance disorder [31]. Lower GCS scores are also associated with the incidence of VAP in TBI patients [32, 38], which may be related to the fact that patients with moderate and severe TBI require open surgery, and respiratory support, and are susceptible to urinary tract infection. A single-center prospective cohort study including 900 patients found that lower GCS scores and higher APACHE (Acute Physiology and Chronic Health Evaluation) II scores are independent risk factors for secondary sepsis in TBI patients after operation [39].

Furthermore, the blood calcium level is included in many machine learning models for sepsis prediction [20]. Critical diseases themselves are correlated with decreased serum total calcium and ionic calcium levels, and hypocalcemia also worsens with the increase in infection severity [40], which may be due to the increased sensitivity of parathyroid cells to blood calcium concentration [41]. This indicates the role of blood calcium levels in predicting the risk of sepsis among infected patients.

With an AUC of greater than 0.7, our prediction model demonstrated favorable prediction efficiency and filled the gap in tools for predicting sepsis in TBI patients admitted to the ICU. Our prediction model enables clinicians to identify the risk of secondary sepsis in TBI patients at an early stage and develop targeted treatment plans according to risk factors, thus reducing the incidence of sepsis and improving the prognosis of patients.

However, this study has several limitations. First of all, our model has not been verified in an external cohort, and we will carry out further research in the future. Secondly, due to the limited types of variables in the public database, some variables of interest, such as cerebrospinal fluid examination and brain imaging data, were not included in the study.

## Conclusion

AKI, anemia, invasive ventilation, GCS score, lactic acid, and serum calcium levels are significant predictors. We have developed a nomogram model for predicting secondary sepsis in TBI patients admitted to ICU. The model has a favorable prediction performance and can provide useful predictive information for clinical decision-making.

## Data Availability

he datasets generated and/or analyzed during the current study are available in the MIMIC-IV 2.0 database (https://physionet.org/content/mimiciv/2.0/).

## Declarations

**Ethics approval and consent to participate**

The study was an analysis of anonymized publicly available databases with preexisting institutional review board approval.
